# Characterization of Acid‐Soluble Collagen From By‐Product Bones of European Sea Bass (*Dicentrarchus labrax*) and Common Carp (*Cyprinus carpio*)

**DOI:** 10.1002/fsn3.70059

**Published:** 2025-04-09

**Authors:** Çiğdem Dikel, Yasemen Yanar

**Affiliations:** ^1^ Department of Biotechnology Cukurova University Adana Türkiye; ^2^ Faculty of Fisheries, Department of Seafood Processing Technology Cukurova University Adana Türkiye

**Keywords:** acid‐soluble collagen, by‐product, characterization, common carp bone, sea bass bone

## Abstract

Cultured European sea bass ( *Dicentrarchus labrax* ) and common carp ( *Cyprinus carpio* ) bones are crucial sources of fish collagen and can be used as a substitute for mammalian collagen. In this study, acid‐soluble collagen (ASC) were extracted from European sea bass ( *Dicentrarchus labrax* ; ASC‐S) and common carp ( *Cyprinus carpio* ; ASC‐C). Based on dry weight, collagen extracted from sea bass and common carp bones using acid treatments was 4.06% and 5.09%, respectively. Collagen extracted from common carp bones was higher than from sea bass bones (*p* < 0.05). Glycine is the primary amino‐acid in both collagen, whereas proline, alanine, hydroxyproline, arginine and glutamic acid are all rather abundant. In addition, FTIR spectra revealed that the amide A, B, amide I, II, and III peaks of collagens were compatible and highly comparable with each other. According to research using SEM, both collagens have a fibrous structure and are porous. The collagen from the bones of sea bass and common carp was found to have a denaturation temperature of 32.17°C and 34.76°C, respectively, which is greater than that of the majority of other fish species. According to the findings of the X‐Ray Diffraction (XRD) examination, the two collagens kept their helical configurations. These findings suggest that a fish's living environment—whether it is freshwater or saltwater—has no direct impact on the qualities of its collagen, and that fish collagen may be used as a substitute for collagen derived from terrestrial animals in the food packaging, nutraceutical, and pharmaceutical sectors.

## Graphical abstract

Extraction of collagen from sea bass and common carp bone waste is a cost‐effective and sustainable way to utilize valuable protein by reducing waste disposal costs. Both collagens contain glycine as the main amino acid with relatively high content of proline, alanine, hydroxyproline, arginine and glutamic acid. FTIR spectra showed that both groups maintained the triple helical structure, although ASC could self‐aggregate. SEM studies revealed that both collagens exhibited porous and fibrous structures.

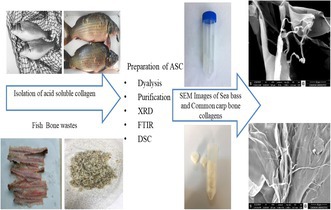

## 1 Introduction

Fish is a perishable food that spoils almost soon after being harvested. To increase the shelf life of fish, multiple processing procedures, such as filleting, salting, packaging, or smoking, must be used (Majidiyan et al. 2022). These processing techniques typically create large amounts of waste—between 30% and 70% of total fish mass (FAO 2021). Fish processing waste refers to the by‐products generated during the processing of fish, such as heads, tails, bones, and viscera. These by‐products are often considered a nuisance and are disposed of in landfills or incinerated, resulting in environmental pollution and economic loss. However, this waste can contain significant amounts of valuable nutrients, such as protein, lipids, and minerals, which can be recovered and used as animal feed, fertilizers, or biofuels. In recent years, there has been an increasing interest in utilizing fish processing waste to produce value‐added products, such as fish oil, hydroxyapatite, gelatin, and collagen. Collagen is a structural protein found in the connective tissue of animals and is an important component in the cosmetics and food industries (Giannetto et al. 2020). Fish collagen has gained attention due to its high bioavailability, low immunogenicity, and good gel‐forming properties (Xu et al. 2018). Studies have shown that fish collagen can help to improve skin health by increasing hydration and reducing the appearance of fine lines and wrinkles. It may also help to stimulate collagen production, which is important for maintaining the skin's elasticity and firmness (Sibilla et al. 2015). In addition to its effects on skin health, fish collagen has been shown to have anti‐inflammatory properties, which may make it useful in the treatment of conditions such as osteoarthritis and rheumatoid arthritis (Luo et al. 2022).

Türkiye has a rapidly growing aquaculture industry, with a focus on fish production. According to the Turkish Statistical Institute (Turkish Statistical Institute 2020), Türkiye produces around 421,000 tons of fish and shellfish, which includes species such as sea bass, sea bream, and trout European sea bass ( *Dicentrarchus labrax* ) production in Türkiye increased from 17,877 tons in 2000 to 155,151 tons in 2020 (Turkish Statistical Institute 2022). However, there are few studies on the evaluation of European sea bass ( *Dicentrarchus labrax* ) waste.

Common carp ( *Cyprinus carpio* ) is a fish species that is commonly caught in Türkiye as part of the country's fishing activities. Türkiye produces around 39,000 tons of carp, mostly caught in the country's freshwater, such as rivers, lakes, and dam lakes (Turkish Statistical Institute 2020). The edible parts of carp flesh are 27.3%–27.9% of its total body weight (Suhenda and Praseno 2017). By‐products (head, swim bladder, bone, skin, and scales) are formed at a rate of approximately 50%–70% of fish weight during food processing (Liu et al. 2015). These by‐products, which are not used adequately in many places, can be used efficiently to produce value‐added products and prevent environmental pollution (Wang et al. 2014).

It can be predicted that collagen obtained from different fish species differ in terms of molecular composition and functional properties. In addition, it is assumed that the environment and body temperatures affect fish collagen properties. Therefore, in this study, collagen was extracted from the bones of two different fish species, freshwater ( *Cyprinus carpio* ) and saltwater fish ( *Dicentrarchus labrax* ), and characterized and compared with each other.

## 2 Material and Methods

### 2.1 Fish Species

The European sea bass ( *Dicentrarchus labrax* ) and common carp ( *Cyprinus carpio* ) were bought from the neighborhood wholesale market in Adana City, Turkey. After the bones were physically removed, cleaned samples were given a tap water wash. Before being utilized, the bones were sliced into small pieces (1–2 cm long) and kept at −25°C.

### 2.2 Preparation of Collagen From Fish Bone

With a small modification, the technique described by Fatiroi et al. (2023) was used to make the collagens. Every step of the preparation process was carried out at 4°C. The non‐collagenous proteins were extracted from the bones by soaking them in 0.1 N NaOH for 3 days at a sample/alkali solution ratio of 1:10 (w/v). Every day, the NaOH solution was replaced, and then it was cleaned with cold, distilled water. In order to extract collagen from bones, deproteinized bones were decalcified for 5 days at a solid/solution ratio of 1:10 (w/v) with 0.5 M ethylenediaminetetraacetic acid (EDTA)–4Na (pH 7.4). The solution was replaced every 24 h. The decalcified bone samples were placed in a 10% butyl alcohol 1:10 (g bone/ml) solution for 3 days, with a change of solution every 24 h, in order to extract the fat. Collagen was extracted from bone samples using a 0.5 M acetic acid solution over the course of 3 days. Every 24 h, the solution was replaced, and the filtrate was gathered in a different container. Salt precipitation was applied to the mixture of extracts. The collagen was precipitated by adding NaCl (powder) to a final concentration of 2.5 M in the presence of 0.05 M tris (hydroxymethyl) aminomethane, pH 7.0. Centrifugation at 10,000 rpm for an hour was used to separate the resulting precipitate, which was then dissolved in 0.5 M acetic acid. After dialyzing the resultant solution against a 0.1 M acetic acid solution and distilled water, acid‐solubilized collagen, or ASC, was produced.

### 2.3 Characterization of Acid‐Solubilized Collagen

#### 2.3.1 Collagen Yields

Collagen yield was calculated using the dry weight of the material, as specified in the following formula.

$$\text{Collagen yield}\;\left(\frac{g}{100g}\right)=\frac{\text{Weight of lyophilized collagen}}{\text{Initial weight of lyophilized fish skin}}\times 100$$

#### 2.3.2 Ultraviolet Spectra Analysis

Using an Agilent Technologies Cary 100 UV–Vis spectrophotometer, the UV spectra of collagen samples were acquired. At a concentration of 0.2 mg/mL, the samples were dissolved in 0.5 M acetic acid. The UV–Vis spectra of each cleared sample were obtained by measuring its absorbance at various wavelengths (between 200 and 400 nm).

#### 2.3.3 Fourier Transform‐Infrared Spectroscopy

Using the JASCO ATR Pro One Model 6700 FT/IR spectrometer (JASCO International Co. Ltd. Hachioji, Tokyo, Japan), collagens' Fourier transform‐infrared spectroscopy (FTIR) spectra were obtained. Spectra Manager TM II was a cross‐platform software application used for spectrum data analysis (JASCO).

#### 2.3.4 Differential Scanning Calorimetry

Thermal stability of ASC was performed using differential scanning calorimetry (DSC) (Mettler Toledo, model DSC 3, Schwerzenbach, Switzerland). For 2 days, lyophilized collagen samples were stored at 4°C after being gelled with 0.05 M acetic acid at a solid/liquid ratio of 1:40 (w/v). The Mettler Toledo (Schwerzenbach, Switzerland) type DSC three instrument was used for measurement.

#### 2.3.5 X‐Ray Diffraction

Using an X‐ray diffraction (XRD; PANanalytical X'Pert High Score Empyrean 45 kV, 40 mA) at a scanning range of 5° to 45° at a scanning speed of 0.5° min and a step range of 0.02°, the crystal structures of the lyophilized collagen samples were ascertained.

#### 2.3.6 Scanning Electron Microscopy and Elemental Chemical Analysis

Quanta 650 Model (Colombus, Ohio, ABD) was used for scanning electron microscopy (SEM). The samples' surfaces were coated with gold–palladium (Au/Pd) to make them conductive. Additionally, the elemental chemical analysis (EDS) method was used to identify the primary constituents of several superficial zones.

#### 2.3.7 Sodium Dodecyl Sulfate–Polyacrylamide Gel Electrophoresis (SDS–PAGE)

SDS‐PAGE was performed by the method of Laemmli. The samples were mixed with 5% (w/v) SDS and heated at 85°C for 10 min. The mixtures were then centrifuged at 8500 × *g* for 5 min to remove undissolved debris. Solubilized samples were mixed at 1:4 (v/v) ratio with Laemmli (1970), (4X) (0.5 M Tris–HCl, pH 6.8, containing 4% (w/v) SDS, 20% (v/v) glycerol) in the presence of 10% (v/v) 𝛽ME. Samples (40 𝜇g protein) were loaded onto a polyacrylamide gel made of 10% separating gel and 5% stacking gel and subjected to electrophoresis at a constant current of 15 mA/gel, using a Mini‐PROTEAN II unit (Bio‐Rad Laboratories Inc., Richmond, CA, USA). After electrophoresis, gels were fixed with a mixture of 50% (v/v) methanol, 10% (v/v) acetic acid and 0.05% (w/v) CoomassieBlue R‐250 for 2 h. Finally, gels were destained with the mixture of 40% (v/v) methanol and 10% (v/v) acetic acid for overnight. Molecular‐weight protein markers (SeeBlue Plus2 prestained Standard, İnvitrogen Inc., UAS) were used to estimate the molecular weight of proteins. Gels were imaged using a iBright CL750 Imaging Systems (iBright LC 750, İnvitrogen Inc., USA).

#### 2.3.8 Amino Acid Composition

Samples of collagen were hydrolyzed for 24 h at 110°C in 6 N HCl under decreased pressure. An HPLC equipment, the Shimadzu model Nexera‐X2 was used to analyze amino acids. The analysis was performed in two replicates. The retention periods and peak areas of the standards were used to define and calculate amino acids.

#### 2.3.9 Statistical Analysis

The present investigation's findings are presented as mean ± standard deviation. The Statistical Package for Social Sciences (SPSS) (Pallant 2020) was used to conduct the statistical analysis. To find the differences between the groups, one‐way ANOVA was employed. Pair comparisons also employed the *T*‐test. Statistical significance was defined as any *p*‐value less than 0.05 (*p* < 0.05).

## 3 Result and Discussion

### 3.1 Collagen Yield

The yields of ASC extracted from sea bass bone (ASC‐S) and carp (ASC‐C) were 4.06% and 5.09%, respectively, based on dry weight. It was discovered that the collagen production derived from carp bones was greater than that of sea bass bones (*p* < 0.05). Fish species can range greatly in their collagen yields because of a variety of factors, including age, the source of the tissue, and the biology of the species. The collagen yield observed in our investigation is comparable to that of deep‐sea redfish ( *Sebastes mentella* ) bones (6.7%) and *Lutjanus sp*. bones (4.53%; Ramli et al. 2019). It is, however, larger than the recorded yield for dusky grouper ( *Epinephelus marginatus* ) (0.39%), red mullet ( *Mullus barbatus* ), (0.62%), common seabream ( *Pagrus pagrus* ) (0.61%), gilt‐head seabream ( *Sparus aurata* ) (1.13%), shi drum ( *Umbrina cirrosa* ) (0.41%) and Atlantic salmon ( *Salmo salar* ) (0.87%) fish scales (Tziveleka et al. 2022) and lower than the published yields for black carp (15.5%; Jia et al. 2012) and clown featherback fish ( *Chitala ornata* ) skin (16.04%; Le et al. 2020). According to Anand et al. (2013), the acetic acid extraction procedure produced a dry weight basis collagen yield of 7.5% in Australian snapper ( *Pagrus auratus* ) skin and 8% in Asian sea bass ( *Lates calcarifer* ) skin. According to reports, there are differences in these collagen yields depending on the species, age, size, starving state, tissue composition and structure, and extraction technique (Jaziri et al. 2022a, 2022b; Fatiroi et al. 2023; Zhang et al. 2010).

### 3.2 UV–vis Spectrometer

**FIGURE 1:**
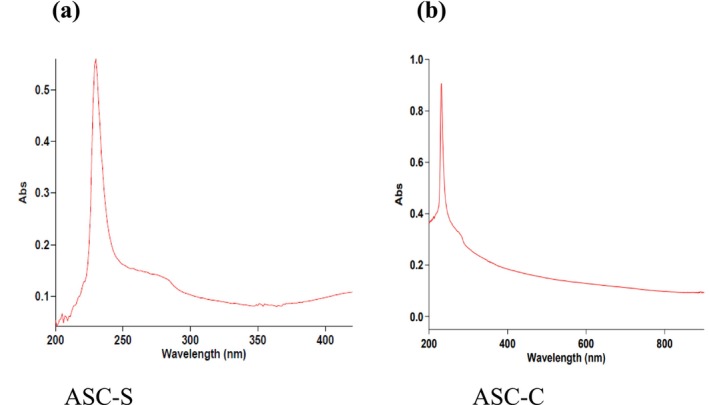
UV–Vis spectra of ASC‐S (Sea bass bone collagen) (a) and ASC‐C (Carp bone collagen) (b).

The greatest absorption maxima of sea bass and carp bone collagens were observed at 232 nm and 230 nm, respectively, as seen in Figure 1. The structural properties of collagen can be better understood by examining its UV–Vis spectra. In the UV–Vis spectrum of fish bone collagen, the absorption maxima at 232 and 230 nm are in agreement with the distinctive absorbance of Type 1 collagen. At 280 nm, protein absorption is often at its highest. Tryptophan and tyrosine are two amino acids that strongly absorb in this wavelength range, and neither of the collagens ASC‐S nor ASC‐C showed an absorption peak at 280 nm (Huang et al. 2011). Collagen isolated from Mesopotamian spiny eel (Göçer et al. 2024), purple‐skin‐ spotted Bigeye Snapper (Oslan et al. 2022), and big fin long barbel catfish (Zhang et al. 2009) showed similar results.

### 3.3 Fourier Transform Infrared Spectroscopy

**FIGURE 2:**
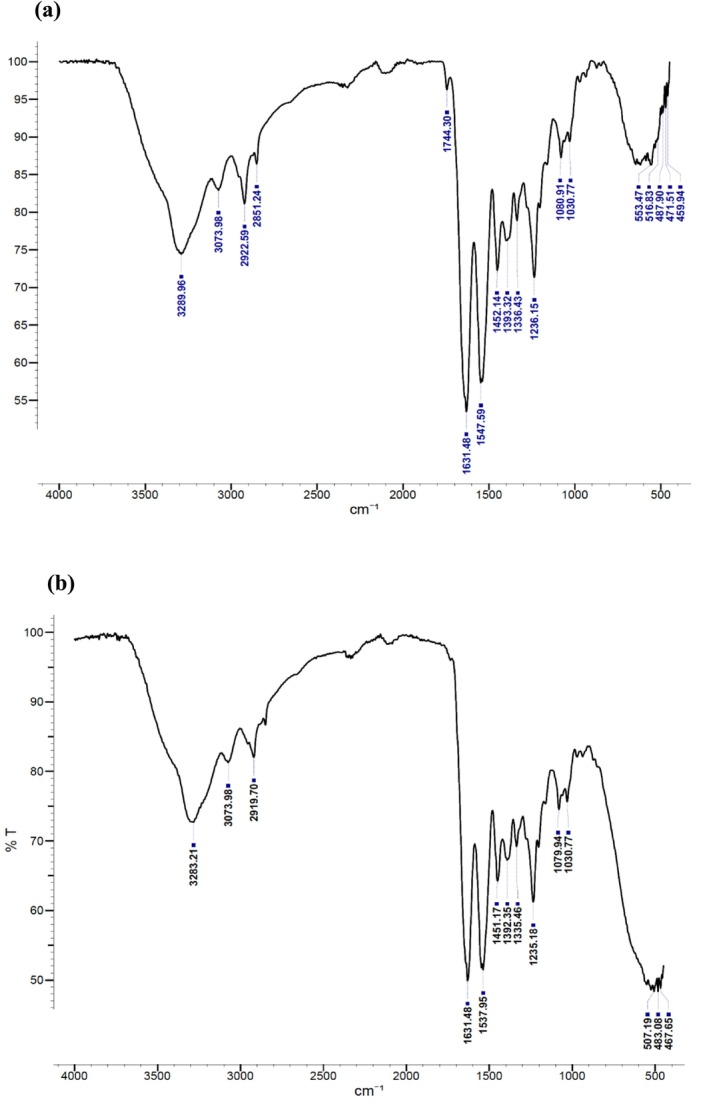
Structure of the ASC‐S (a) and ASC‐C (b) FTIR spectrum.

FTIR spectroscopy offers valuable insights into molecular structures, making it a useful tool for analyzing and determining the secondary structure of proteins (Jiang et al. 2011). In this study, FTIR spectroscopy was utilized to analyze the functional groups and secondary structure of ASCs from ASC‐S and ASC‐C. Figure 2 displays the ASC FTIR spectra from European sea bass and common carp bones. It was discovered that the amide A absorption peaks of ASC‐S and ASC‐C were, respectively, 3289.96 cm^−1^ and 3283.21 cm^−1^. This is associated with stretching vibrations of the N‐H and often takes place in the 3400–3440 cm^−1^ range (Kaewdang et al. 2014). A peptide's N‐H group participates in the creation of hydrogen bonds, which causes amide A to move to a lower frequency. According to Chen et al. (2019), the amide B band's absorption peak, measured at 2958.31 cm^−1^, was observed in collagen extracted from Red Stingray ( *Dasyatis akajei* ) skin. This peak is often seen in the 3080 cm^−1^ range and is associated with the asymmetric stretching of CH₂. The ASC‐C and ASC‐S collagens that we studied have amide B bands at 3078.98 cm^−1^ and 3073.98 cm^−1^, respectively.

The amide I, II, and III regions are closely related to the structure of polypeptides. The amide I band is linked to the C=O stretching vibration along the polypeptide backbone or a hydrogen bond coupled with COO–, with strong absorption in the 1600–1700 cm^−1^ range. This band is regarded as the most critical indicator for determining the secondary structure of proteins (Liu et al. 2014; Pati et al. 2010). As shown in Figure 2, the amide I bands for ASC‐S and ASC‐C is at 1631.48 cm^−1^. This indicates that the proteins have a well‐organized secondary structure with strong hydrogen bonding. It also implies a high degree of molecular order and structural stability. Liu et al. (2014) reported the amide I peak at approximately 1650 cm^−1^ for carp skin collagens.

The amide II band, typically observed in the 1550–1600 cm^−1^ range, is primarily associated with N‐H bending combined with C‐N stretching vibrations. A shift to lower wavelengths in this region indicates the formation of hydrogen bonds. In other words, the amide II band specifies the number of NH groups involved in hydrogen bonding with the adjacent α‐chain; therefore, a lower wavenumber of the amide II band indicates both increased hydrogen bonds by NH groups, and a higher structure order (Woo et al. 2008). The wave numbers of ASC‐S and ASC‐C were found to be 1547.59 cm^−1^ and 1537.95 cm^−1^, respectively. Based on this, the current data suggest that ASC‐C has a higher amount of hydrogen bonding compared to ASC‐S. Muyonga et al. (2004) observed the amide II peak in the 1540–1558 cm^−1^ range for Nile perch skin collagen.

The amide III band is associated with C‐N stretching and N‐H bending and is linked to the triple helical structure of collagen (Woo et al. 2008). In this study, the amide III bands of ASC‐S and ASC‐C were located at wave numbers of, 1236.15 cm^−1^ and 1235.18 cm^−1^, respectively. The results showed that hydrogen bonds were present in both ASC‐S and ASC‐C. Additionally, absorption peaks at 1452.14 cm^−1^ for ASC‐S and 1451.17 cm^−1^ for ASC‐C were observed, corresponding to the pyrolidine ring vibrations of proline and hydroxyproline (Muyonga et al. 2004). The intensity ratio between the amide II band and the 1450 cm^−1^ band has been used to clarify the triple‐helical structure of collagen (Guzzi Plepis et al. 1996). In this study, the absorption ratio between the amide III bands (ASC‐S at 1236.15 cm^−1^, ASC‐C at 1235.18 cm^−1^) and the 1452.14 cm^−1^ (ASC‐S) or 1451.17 cm^−1^ (ASC‐C) bands was approximately 1.1. This confirmed that the triple‐helical structure of both ASC‐S and ASC‐C remained intact, with a high degree of intermolecular organization preserved (Guzzi Plepis et al. 1996).

### 3.4 Differential Scanning Calorimetry

**FIGURE 3:**
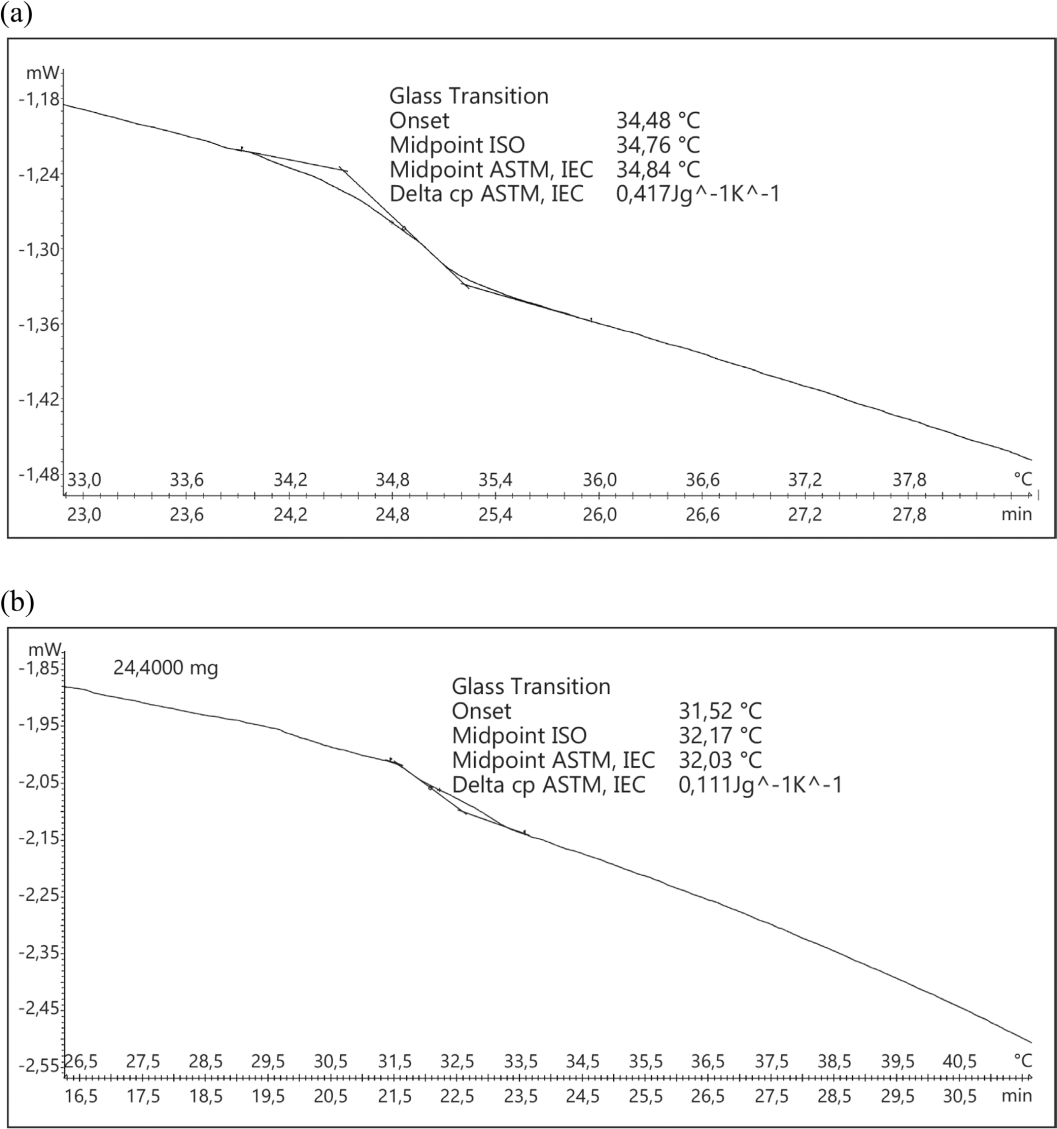
DSC‐*T*_max_ values of acid‐soluble collagen extracted from sea bass bones (ASC‐S) (a) and carp bones (ASC‐C) (b).

Figure 3 displays the maximum transition temperatures (*T*_max_) of ASC that were isolated from ASC‐S and ASC‐C bones and dissolved in 0.5 M acetic acid. The ASC‐S and ASC‐C were found to have *T*_max_ and enthalpy (∆H) values of 32.17°C and 0.111 J/g and 34.76°C and 0.417 J/g, respectively. Collagen's mix of amino acids, especially imino acids, affects its heat stability. Fish collagen has been shown to have a larger imino acid concentration when the thermal denaturation temperature is higher (Chen et al. 2022). In our investigation, we determined that the imino acid (proline + hydroxyproline) contents of ASC‐S and ASC‐C were 27.81% and 27.65%, respectively. These values were found by Montero et al. (1990), while values for the Nile perch, *Salmo irideus*, and *Merluccius merluccius* were published by Muyonga et al. (2004). Our findings indicate that the denaturation temperature and imino acid concentration are positively correlated. *T*_max_ values for various collagen sources have been reported in a number of studies, and it has been hypothesized that *T*_max_ is dependent on fish species, age, age‐related changes in habitat temperature and environment, and season (Chuaychan et al. 2015; Liu et al. 2018; Thuy and Minh 2012; Tang et al. 2015).

### 3.5 X‐Ray Diffraction

**FIGURE 4:**
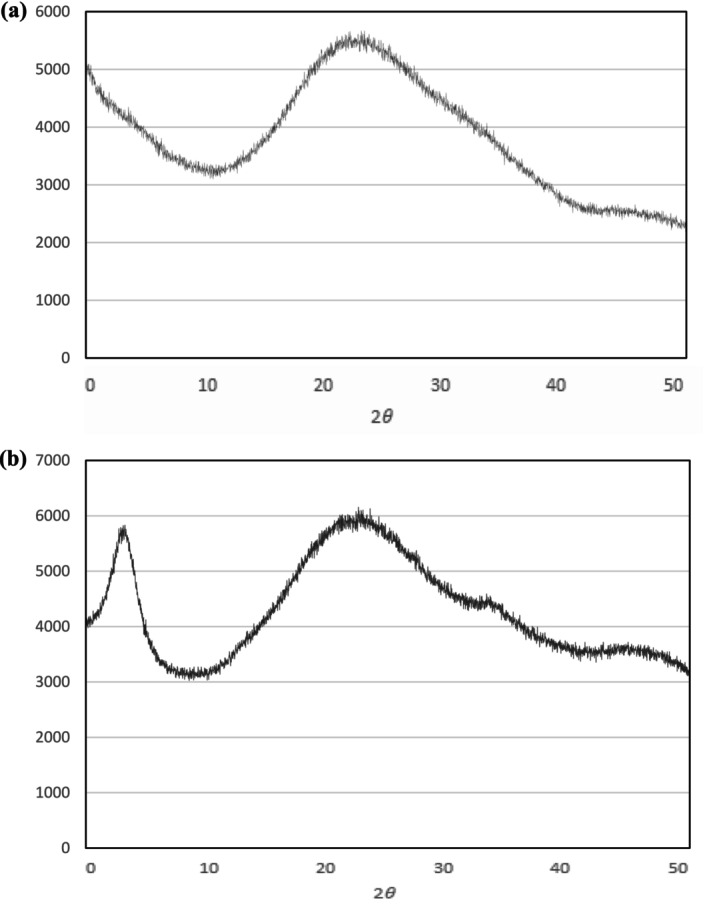
X‐Ray diffraction spectra of acid‐soluble collagen extracted from European sea bass bones (ASC‐S) (a) and common carp bones (ASC‐C) (b).

This method studies collagen's atom‐by‐atom arrangement using X‐rays, which can reveal details about the material's crystal structure. The XRD curve for ASC‐C and ASC‐S exhibits distinct refractive peaks at diffraction angles (2θ) of approximately 7.23° and 21.53° for ASC‐S and 7.57° and 23.34° for ASC‐C, respectively, as seen in Figure 4. The triple helix structure of collagen is associated with the first sharp peak, and the spacing between the chains is shown by the second wide peak (Bella et al. 1994). These findings verify that neither collagen is denatured and that it still has its triple helix structure. Comparable outcomes were seen for the collagen of tiapia *(Oreochromis niloticus)* scales (Chen et al. 2016), the bone collagen of lizardfish ( *Saurida tumbil* ) (Jaziri et al. 2022a), the bone collagen of unicornfish ( *Naso reticulatus* Randall) (Fatiroi et al. 2023), the collagen of Atlantic cod, and the collagen of Atlantic salmon skin (Alves et al. 2017).

### 3.6 Scanning Electron Microscopy and Elemental Chemical Analysis

**FIGURE 5:**
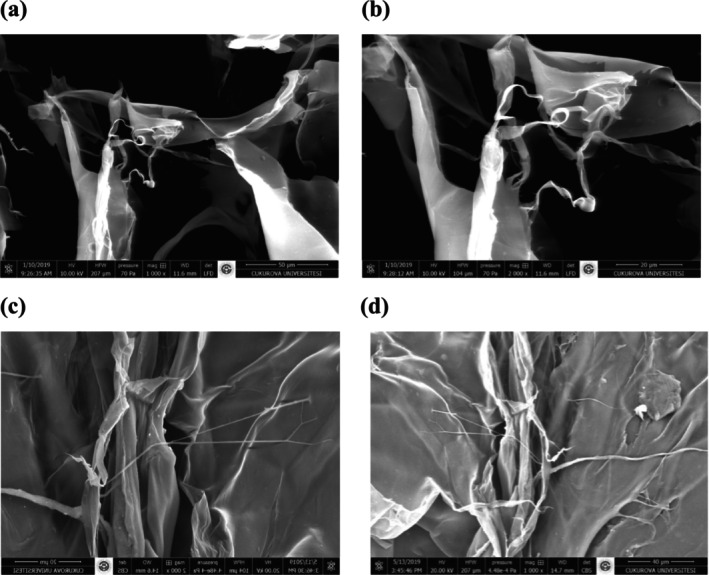
SEM images of acid soluble collagen from sea bass bones (a, b); carp bones (c, d). a, ×1000; b, ×2000; c, ×1000; d, ×2000.

The morphological structures of the extracted lyophilized collagen (ASC‐S and ASC‐C) were visualized via SEM under ×1000 and ×2000 magnifications (Figure 5A,B). When viewed with the naked eye, both collagens appeared as a soft white sponge with a loose and porous structure. However, in SEM images, the multilayer exhibited a partially wrinkled, sheet‐like structure, possibly due to dehydration during lyophilization, along with irregular, complex collagen fibrils connected by randomly wound filaments. However, irregular and tubular structures have also been observed. Similarly, Ramanathan et al. (2014) observed a layer‐by‐layer structure of ASC extracted from *Arothron stellatus* skin, and this is related to the intertwining of collagen fibers. Furthermore, Rizk and Mostafa (2016), Tziveleka et al. (2017), Rodrigues et al. (2010), and Pal et al. (2015) stated that SEM images of the collagens they extracted had smooth or slightly wrinkled surfaces or sheet‐like structures.

**FIGURE 6:**
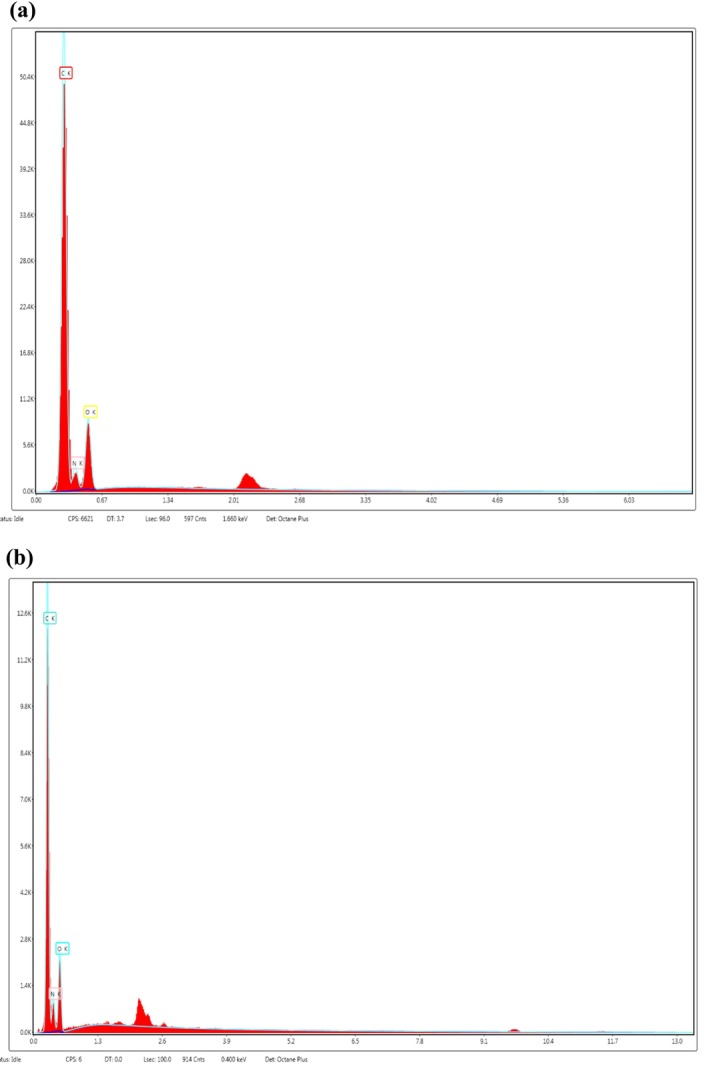
EDS images of acid soluble collagen from sea bass bones (a); carp bones (b).

The characterization of collagen using SEM/EDS is crucial in establishing its purity. Figure 6A,B EDS spectra of ASC‐S and ASC‐C revealed the presence of low‐intensity gold (Au) peaks emanating from the metallic coating, as well as carbon (C), oxygen (O), and nitrogen (N). In this instance, the great purity of the resulting collagens was demonstrated by the fact that both collagens comprised just C, O, and N components, and that the quantities of these elements were C>O>N, respectively. It has been noted that processing conditions, including the use of nano‐grinding, can lead to partial degradation of collagen by affecting its solubility and structural integrity. This degradation may cause collagen fibers to appear less organized or fragmented in SEM images (Li et al. 2020).

### 3.7 Sodium Dodecyl Sulfate–Polyacrylamide Gel Electrophoresis (SDS–PAGE)

**FIGURE 7:**
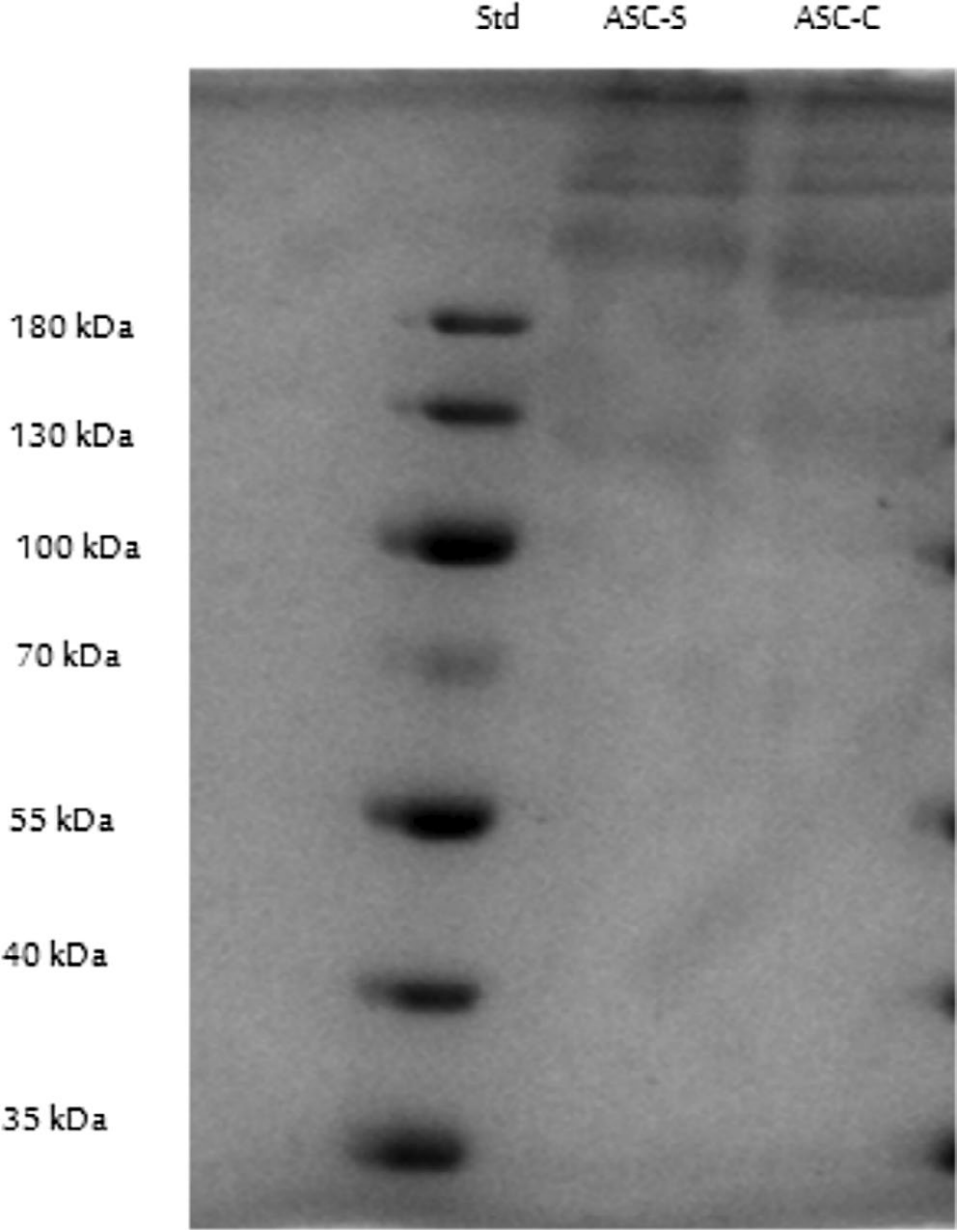
SDS‐PAGE profile of seabass (ASC‐S) and common carp bones(ASC‐C) collagen.

Sodium dodecyl sulfate‐polyacrylamide gel electrophoretic (SDS‐PAGE) patterns of ASCs from seabass and common carp bones are shown in Figure 7. Three distinctive chains were detected in both ASCs from seabass and common carp bones: 2 α bands (α1, upper; α2, lower) with molecular weights of approximately 100–130 kDa, and their β‐cross linked components, with a molecular weight of above 180 kDa. This is in accordance with those of collagens from most other fish species previously reported such as Catfish (Abbas 2022), Amazonian freshwater fish pirarucu (Carpio et al. 2023), cuckoo ray, common Atlantic grenadier lantern shark, catshark, small‐ spotted catshark (Sotelo et al. 2016), brown banded bamboo shark (Kittiphattanabawon et al. 2010), striped catfish (Singh et al. 2011), brown backed toadfish (Senaratne et al. 2006) and is typical of type I collagen (Ahn et al. 2021). Fish skin and bone have been reported to contain type I collagen as the major collagen (Liu and Huang 2016; Fatiroi et al. 2023; Jaziri et al. 2022a; Guiry et al. 2016; Wijaya 2021).

### 3.8 Amino Acid Analysis

Table 1 displays the identical amino acid contents of ASCs recovered from the bones of European sea bass (ASC‐S) and common carp (ASC‐C), with the exception of glutamic acid (*p* > 0.05). In ASC‐S and ASC‐C, glycine made up 27.54% and 29.46% of the total amino acid content, respectively.

**TABLE 1:** Amino acid compositions of ASC‐S and ASC‐C.

Amino acid	ASC‐S (g/100 g protein)	ASC‐C (g/100 g protein)
Aspartic acid	4.48 ± 0.18	4.93 ± 0.23
Glutamic acid	5.85 ± 0.03*	7.15 ± 0.34
Serine	4.56 ± 0.22	3.42 ± 0.22
Glycine	27.54 ± 0.14	29.46 ± 0.25
Arginine	6.66 ± 0.10	6.34 ± 0.13
Alanine	8.29 ± 0.15	8.57 ± 0.07
Tyrosine	0.71 ± 0.03	0.42 ± 0.01
Cysteine	ND	ND
Valin	0.74 ± 0.03	0.60 ± 0.06
Methionine	0.68 ± 0.06	0.97 ± 0.07
Tryptophan	ND	ND
Phenylalanine	3.00 ± 0.13	2.85 ± 0.04
Leucine	2.55 ± 0.09	2.17 ± 0.15
Lysine	4.57 ± 0.11	3.75 ± 0.29
Hydroxyproline	14.72 ± 0.20	15.16 ± 0.56
Proline	13.09 ± 0.15	12.49 ± 0.07
Total imino acid (hydroxyproline + proline)	27.81 ± 0.34	27.65 ± 0.50

*Note:* ± indicates the standard deviation. *Statistical difference at the *p* < 0.05. Level was determined between the two groups. (*n* = 2).

The three alpha chains that make up collagen are coiled on top of one another and include sequences, with the amino acid sequences repeating in the form of Glycine‐X‐Y. Put another way, according to Liu et al. (2018), glycine constitutes around one‐third of the amino acids in collagen and is present at every third position in the peptide chain. Our study's two collagen samples attest to this. Compared to other collagens, tryptophan and cystine were absent, while extremely low quantities of other amino acids like tyrosine, valine, and methionine were discovered (Jongjareonrak et al. 2005; Liu and Huang 2016; Koliada and Plavan 2015).

The stability and strength of the triple helix of collagen are dependent on the quantity of imino acids, namely proline and hydroxyproline. Specifically, through the formation of interchain hydrogen bonds, hydroxyproline is essential in maintaining the stability of the triple helix (Xu et al. 2019). Proline plus hydroxyproline made up the total quantity of imino acids, which was determined to be 27.81% and 27.65% for ASC‐S and ASC‐C, respectively. These results were statistically identical (*p* > 0.05). These values were reported in the study by Montero et al. (1990) for *Salmo irideus* and *Merluccius merluccius* , and by Muyonga et al. (2004) for Nile perch. They were discovered, nonetheless, in proximity to the tilapia reported value of 25.4% (Grossman and Bergman 1992). The relationship between the imino acid concentration and the thermal stability of collagen is also well‐established; as noted by Truong et al. (2021), Kawaguchi et al. (2011), and Kiew and Mashitah (2013), denaturation temperatures of collagens rise with imino acid content. According to Akita et al. (2020), collagens that are isolated from animals that live in cold climates are said to have lower melting points and lower levels of thermal stability due to their low proline and hydroxyproline concentrations. Additionally, it has been reported that one of the primary causes of the high denaturation temperature in commercial collagen derived from the bovine tendon is its high concentration of proline, hydroxyproline, and alanine (Le et al. 2020). Since sea bass and carp are warm‐climate fish, the imino acid concentration of both collagens obtained in our study is predicted for them. This helps to explain why the denaturation temperatures of ASC‐S and ASC‐C are greater than those of cold‐climate fish (32.17°C and 34.76°C, respectively).

## 4 Conclusions

Collagen extraction from fish processing wastes is a cost‐effective and sustainable way to utilize these underutilized resources, as it allows for the recovery of valuable protein while reducing waste disposal costs. Fish bone contains collagen, although in lower amounts than fish skin. However, this by‐product is more abundant than skin and could, therefore, be an important source of collagen. In this study, ASC extraction and characterization were performed from the European sea bass ( *Dicentrarchus labrax* ) and common carp ( *Cyprinus carpio* ) bones, which are abundant in Türkiye. It was shown that sea bass and carp bone wastes are a source of Type 1 collagen. Its porous and irregular microstructure shows its potential for use as a biomaterial. Therefore, future studies should focus on the use of collagen from sea bass and carp bone by‐products as an alternative to mammalian collagen and as a valuable and environmentally friendly material in cosmetic, biopharmaceutical, biomaterial, and food applications. It has also been observed that the environment in which a fish lives, such as seawater or freshwater, has no direct effect on collagen properties.

## Author Contributions

**Çiğdem Dikel** and **Yasemen Yanar:** concept. **Yasemen Yanar:** sample collection. **Çiğdem Dikel** and **Yasemen Yanar:** methodology and applications. **Çiğdem Dikel** and **Yasemen Yanar:** literature review, data collection or processing. **Çiğdem Dikel** and **Yasemen Yanar:** writing.

## Conflicts of Interest

The authors declare no conflicts of interest.

## Data Availability

Data are made available upon reasonable request by contacting the corresponding author.

## References

[fsn370059-bib-0001] Abbas, A. A. 2022. “Functional Properties of Collagen Extracted From Catfish (*Silurus triostegus*) Bone.” Journal of Biotechnology Research Center 16, no. 2: 104–116. 10.3390/foods11050633 PMC8909090 35267266

[fsn370059-bib-0002] Ahn, H. , D. J. Gong , H. H. Lee , et al. 2021. “Mechanical Properties of Porcine and Fish Skin‐Based Collagen and Conjugated Collagen Fibers.” Polymers 13, no. 13: 2151. 34209976 10.3390/polym13132151 PMC8271417

[fsn370059-bib-0003] Akita, M. , Y. Nishikawa , Y. Shigenobu , et al. 2020. “Correlation of Proline, Hydroxyproline and Serine Content, Denaturation Temperature and Circular Dichroism Analysis of Type I Collagen With the Physiological Temperature of Marine Teleosts.” Food Chemistry 329: 126775. 32512387 10.1016/j.foodchem.2020.126775

[fsn370059-bib-0004] Alves, A. L. , A. L. Marques , E. Martins , T. H. Silva , and R. L. Reis . 2017. “Cosmetic Potential of Marine Fish Skin Collagen.” Cosmetics 4, no. 4: 39.

[fsn370059-bib-0005] Anand, S. , S. Kamath , L. Chuang , S. Kasapis , and A. L. Lopata . 2013. “Biochemical and Thermo‐Mechanical Analysis of Collagen From the Skin of Asian Sea Bass (*Lates calcarifer*) and Australasian Snapper (*Pagrus auratus*), an Alternative for Mammalian Collagen.” European Food Research and Technology 236: 873–882.

[fsn370059-bib-0006] Bella, J. , M. Eaton , B. Brodsky , and H. M. Berman . 1994. “Crystal and Molecular Structure of a Collagen‐Like Peptide at 1.9 Å Resolution.” Science 266, no. 5182: 75–81. 7695699 10.1126/science.7695699

[fsn370059-bib-0007] Carpio, K. C. R. , R. S. Bezerra , T. B. Cahú , et al. 2023. “Extraction and Characterization of Collagen From the Skin of Amazonian Freshwater Fish Pirarucu.” Brazilian Journal of Medical and Biological Research 56: e12564. 37194834 10.1590/1414-431X2023e12564 PMC10242699

[fsn370059-bib-0008] Chen, J. , J. Li , Z. Li , et al. 2019. “Physicochemical and Functional Properties of Type I Collagens in Red Stingray (*Dasyatisa kajei*) Skin.” Marine Drugs 17, no. 10: 558. 31569390 10.3390/md17100558 PMC6835876

[fsn370059-bib-0009] Chen, J. , L. Li , R. Yi , N. Xu , R. Gao , and B. Hong . 2016. “Extraction and Characterization of Acid‐Soluble Collagen From Scales and Skin of Tilapia (*Oreochromis niloticus*).” LWT‐ Food Science and Technology 66: 453–459.

[fsn370059-bib-0010] Chen, L. , G. Cheng , S. Meng , and Y. Ding . 2022. “Collagen Membrane Derived From Fish Scales for Application in Bone Tissue Engineering.” Polymers 14, no. 13: 2532. 35808577 10.3390/polym14132532 PMC9269230

[fsn370059-bib-0011] Chuaychan, S. , S. Benjakul , and H. Kishimura . 2015. “Characteristics of Acid‐and Pepsin‐Soluble Collagens From Scale of Seabass (*Lates calcarifer*).” LWT‐Food Science and Technology 63, no. 1: 71–76.

[fsn370059-bib-0012] FAO (Food and Agriculture Organization of the United Nations) . 2021. “Fish By‐Products Utilization, Getting More Benefits from Fish Processing Food Loss and Waste in Fish Value Chains.”

[fsn370059-bib-0013] Fatiroi, N. S. , A. A. Jaziri , R. Shapawi , R. A. M. Mokhtar , W. N. M. Noordin , and N. Huda . 2023. “Biochemical and Microstructural Characteristics of Collagen Biopolymer From Unicornfish (*Naso reticulatus* Randall, 2001) Bone Prepared With Various Acid Types.” Polymers 15, no. 4: 1054. 36850337 10.3390/polym15041054 PMC9964761

[fsn370059-bib-0015] Giannetto, A. , E. Esposito , M. Lanza , et al. 2020. “Protein Hydrolysates From Anchovy (*Engraulis encrasicolus*) Waste: In Vitro and In Vivo Biological Activities.” Marine Drugs 18, no. 2: 86. 32012959 10.3390/md18020086 PMC7074155

[fsn370059-bib-0017] Göçer, M. , Y. Yanar , and M. Aydın . 2024. “Comprehensive Analysis of Collagen: Unveiling the Distinctive Characteristics and Amino Acid Profiles From Skin and Bone of Mesopotamian Spiny Eel, *Mastacembelus mastacembelus* (Banks & Solander, 1794).” GSC Advanced Research and Reviews 18, no. 1: 222–234.

[fsn370059-bib-0018] Grossman, S. , and M. Bergman . 1992. U.S. Patent No. 5,093,474. U.S. Patent and Trademark Office.

[fsn370059-bib-0019] Guiry, E. J. , P. Szpak , and M. P. Richards . 2016. “Effects of Lipid Extraction and Ultrafiltration on Stable Carbon and Nitrogen Isotopic Compositions of Fish Bone Collagen.” Rapid Communications in Mass Spectrometry 30, no. 13: 1591–1600. 27321847 10.1002/rcm.7590

[fsn370059-bib-0020] Guzzi Plepis, A. M. D. , G. Goissis , and D. K. Das‐Gupta . 1996. “Dielectric and Pyroelectric Characterization of Anionic and Native Collagen.” Polymer Engineering & Science 36, no. 24: 2932–2938.

[fsn370059-bib-0021] Huang, Y. R. , C. Y. Shiau , H. H. Chen , and B. C. Huang . 2011. “Isolation and Characterization of Acid and Pepsin‐Solubilized Collagens From the Skin of Balloon Fish (*Diodon holocanthus*).” Food Hydrocolloids 25, no. 6: 1507–1513.

[fsn370059-bib-0022] Jaziri, A. A. , R. Shapawi , R. A. M. Mokhtar , W. N. M. Noordin , and N. Huda . 2022a. “Biochemical Analysis of Collagens From the Bone of Lizardfish (*Saurida tumbil* Bloch, 1795) Extracted With Different Acids.” PeerJ 10: e13103. 35310170 10.7717/peerj.13103 PMC8932308

[fsn370059-bib-0023] Jaziri, A. A. , R. Shapawi , R. A. M. Mokhtar , W. N. M. Noordin , and N. Huda . 2022b. “Physicochemical and Microstructural Analyses of Pepsin‐Soluble Collagens Derived From Lizardfish (*Saurida Tumbil* Bloch, 1795) Skin, Bone and Scales.” Gels 8, no. 8: 471. 36005071 10.3390/gels8080471 PMC9407154

[fsn370059-bib-0024] Jia, Y. , H. Wang , H. Wang , Y. Li , M. Wang , and J. Zhou . 2012. “Biochemical Properties of Skin Collagens Isolated From Black Carp (*Mylopharyngodon piceus*).” Food Science and Biotechnology 21: 1585–1592.

[fsn370059-bib-0025] Jiang, Y. , C. Li , X. Nguyen , et al. 2011. “Qualification of Ftir Spectroscopic Method for Protein Secondary Structural Analysis.” Journal of Pharmaceutical Sciences 100, no. 11: 4631–4641. 10.1002/jps.22686. 21713773

[fsn370059-bib-0026] Jongjareonrak, A. , S. Benjakul , W. Visessanguan , and M. Tanaka . 2005. “Isolation and Characterization of Collagen From Bigeye Snapper (*Priacanthus macracanthus*) Skin.” Journal of the Science of Food and Agriculture 85, no. 7: 1203–1210.

[fsn370059-bib-0027] Kaewdang, O. , S. Benjakul , T. Kaewmanee , and H. Kishimura . 2014. “Characteristics of Collagens From the Swim Bladders of Yellowfin Tuna (Thunnus Albacares).” Food Chemistry 155: 264–270. 24594184 10.1016/j.foodchem.2014.01.076

[fsn370059-bib-0028] Kawaguchi, Y. , E. Kondo , N. Kitamura , et al. 2011. “In Vivo Effects of Isolated Implantation of Salmon‐Derived Cross Linked Atelocollagen Sponge Into an Osteochondral Defect.” Journal of Materials Science: Materials in Medicine 22, no. 2: 397–404. 21259035 10.1007/s10856-010-4215-1

[fsn370059-bib-0029] Kiew, P. L. , and M. D. Mashitah . 2013. “Isolation and Characterization of Collagen From the Skin of Malaysian Catfish (Hybrid *clarias Sp*.).” Journal of Korean Society for Applied Biological Chemistry 56: 441–450.

[fsn370059-bib-0030] Kittiphattanabawon, P. , S. Benjakul , W. Visessanguan , and F. Shahidi . 2010. “Isolation and Characterization of Collagen From the Cartilages of Brownbanded Bamboo Shark (*Chiloscyllium punctatum*) and Blacktip Shark (*Carcharhinus limbatus*).” LWT‐Food Science and Technology 43, no. 5: 792–800.

[fsn370059-bib-0031] Koliada, M. , and V. Plavan . 2015. “Problems of Efficient Processing and Use of Collagen‐Containing Materials.” Pure and Applied Chemistry 87, no. 1: 43–49.

[fsn370059-bib-0032] Laemmli, U. K. 1970. “Cleavage of Structural Proteins During the Assembly of the Head of Bacteriophage T4.” Nature 227, no. 5259: 680–685. 5432063 10.1038/227680a0

[fsn370059-bib-0033] Le, T. M. T. , V. M. Nguyen , T. T. Tran , K. Takahashi , and K. Osako . 2020. “Comparison of Acid‐Soluble Collagen Characteristic From Three Important Freshwater Fish Skins in Mekong Delta Region, Vietnam.” Journal of Food Biochemistry 44, no. 9: e13397. 32713023 10.1111/jfbc.13397

[fsn370059-bib-0034] Li, J. , T. Yin , S. Xiong , et al. 2020. “Mechanism on Releasing and Solubilizing of Fish Bone Calcium During Nano‐Milling.” Journal of Food Process Engineering 43, no. 4: e13354. 10.1111/jfpe.13354.

[fsn370059-bib-0035] Liu, D. , X. Zhang , T. Li , et al. 2015. “Extraction and Characterization of Acid‐and Pepsin‐Soluble Collagens From the Scales, Skins and Swim‐Bladders of Grass Carp (*Ctenopharyn godonidella*).” Food Bioscience 9: 68–74.

[fsn370059-bib-0036] Liu, D. , P. Zhou , T. Li , and J. M. Regenstein . 2014. “Comparison of Acid‐Soluble Collagens From the Skins and Scales of Four Carp Species.” Food Hydrocolloids 41: 290–297.

[fsn370059-bib-0037] Liu, H. , and K. Huang . 2016. “Structural Characteristics of Extracted Collagen From Tilapia (*Oreochromis mossambicus*) Bone: Effects of Ethylenediaminetetraacetic Acid Solution and Hydrochloric Acid Treatment.” International Journal of Food Properties 19, no. 1: 63–75.

[fsn370059-bib-0038] Liu, Y. , D. Ma , Y. Wang , and W. Qin . 2018. “A Comparative Study of the Properties and Self‐Aggregation Behavior of Collagens From the Scales and Skin of Grass Carp (*Ctenopharyn godonidella*).” International Journal of Biological Macromolecules 106: 516–522. 28801096 10.1016/j.ijbiomac.2017.08.044

[fsn370059-bib-0039] Luo, X. , W. Liu , M. Zhao , et al. 2022. “A Novel Atlantic Salmon (*Salmo salar*) Bone Collagen Peptide Delays Osteoarthritis Development by Inhibiting Cartilage Matrix Degradation and Anti‐Inflammatory.” Food Research International 162: 112148. 36461366 10.1016/j.foodres.2022.112148

[fsn370059-bib-0040] Majidiyan, N. , M. Hadidi , D. Azadikhah , and A. Moreno . 2022. “Protein Complex Nanoparticles Reinforced With Industrial Hemp Essential Oil: Characterization and Application for Shelf‐Life Extension of Rainbow Trout Fillets.” Food Chemistry: X 13: 100202. 35499007 10.1016/j.fochx.2021.100202 PMC9039897

[fsn370059-bib-0041] Montero, P. , J. Borderias , J. Turnay , and M. A. Leyzarbe . 1990. “Characterization of Hake (Merluccius Merluccius L.) and Trout (Salmo Irideus Gibb) Collagen.” Journal of Agricultural and Food Chemistry 38, no. 3: 604–609.

[fsn370059-bib-0042] Muyonga, J. H. , C. G. B. Cole , and K. G. Duodu . 2004. “Characterisation of Acid Soluble Collagen From Skins of Young and Adult Nile Perch (*Lates niloticus*).” Food Chemistry 85, no. 1: 81–89.

[fsn370059-bib-0043] Oslan, S. N. H. , R. Shapawi , R. A. M. Mokhtar , W. N. M. Noordin , and N. Huda . 2022. “Characterization of Acid‐and Pepsin‐Soluble Collagen Extracted From the Skin of Purple‐Spotted Bigeye Snapper.” Gels 8, no. 10: 665. 36286166 10.3390/gels8100665 PMC9602141

[fsn370059-bib-0044] Pal, G. K. , T. Nidheesh , and P. V. Suresh . 2015. “Comparative Study on Characteristics and In Vitro Fibril Formation Ability of Acid and Pepsin Soluble Collagen From the Skin of Catla (*Catla catla*) and Rohu (*Labeo rohita*).” Food Research International 76: 804–812. 28455066 10.1016/j.foodres.2015.07.018

[fsn370059-bib-0045] Pallant, J. 2020. SPSS Survival Manual: A Step by Step Guide to Data Analysis Using IBM SPSS. Routledge.

[fsn370059-bib-0046] Pati, F. , B. Adhikari , and S. Dhara . 2010. “Isolation and Characterization of Fish Scale Collagen of Higher Thermal Stability.” Bioresource Technology 101, no. 10: 3737–3742. 20116238 10.1016/j.biortech.2009.12.133

[fsn370059-bib-0047] Ramanathan, G. , S. Singaravelu , M. D. Raja , S. S. Sobhana , and U. T. Sivagnanam . 2014. “Extraction and Characterization of Collagen From the Skin of *Arothron stellatus* Fish—A Novel Source of Collagen for Tissue Engineering.” Journal of Biomaterials and Tissue Engineering 4, no. 3: 203–209.

[fsn370059-bib-0048] Ramli, L. , H. Natsir , S. Dali , and S. Danial . 2019. “Collagen Extraction From Bone of *Lutjanus Sp*. and Toxicity Assay.” Jurnal Akta Kimia Indonesia (Indonesia Chimica Acta) 12, no. 1: 67–72.

[fsn370059-bib-0049] Rizk, M. A. , and N. Y. Mostafa . 2016. “Extraction and Characterization of Collagen From Buffalo Skin for Biomedical Applications.” Oriental Journal of Chemistry 32, no. 3: 1601–1609.

[fsn370059-bib-0050] Rodrigues, F. T. , V. C. Martins , and A. M. Plepis . 2010. “Porcine Skin as a Source of Biodegradable Matrices: Alkaline Treatment and Glutaraldehyde Crosslinking.” Polímeros 20: 92–97.

[fsn370059-bib-1001] Senaratne, L. S. , P. J. Park , and S. K. Kim . 2006. “Isolation and Characterization of Collagen From Brown Backed Toadfish (*Lagocephalus gloveri*) Skin.” Bioresource Technology 97, no. 2: 191–197. 15964191 10.1016/j.biortech.2005.02.024

[fsn370059-bib-0051] Sibilla, S. , M. Godfrey , S. Brewer , A. Budh‐Raja , and L. Genovese . 2015. “An Overview of the Beneficial Effects of Hydrolysed Collagen as a Nutraceutical on Skin Properties: Scientific Background and Clinical Studies.” Open Nutraceuticals Journal 8, no. 1: 29–42.

[fsn370059-bib-0052] Singh, P. , S. Benjakul , S. Maqsood , and H. Kishimura . 2011. “Isolation and Characterisation of Collagen Extracted From the Skin of Striped Catfish (*Pangasianodon hypophthalmus*).” Food Chemistry 124, no. 1: 97–105.

[fsn370059-bib-0053] Sotelo, C. G. , M. B. Comesaña , P. R. Ariza , and R. I. Pérez‐Martín . 2016. “Characterization of Collagen From Different Discarded Fish Species of the West Coast of the Iberian Peninsula.” Journal of Aquatic Food Product Technology 25, no. 3: 388–399.

[fsn370059-bib-0054] Suhenda, N. , and O. Praseno . 2017. “Karakteristik daging ikan mas (Cyprinus carpio) yang diberi pakan dengan kadar lemak yang berbeda.” Jurnal Penelitian Perikanan Indonesia 6, no. 1: 13–18.

[fsn370059-bib-0055] Tang, L. , S. Chen , W. Su , W. Weng , K. Osako , and M. Tanaka . 2015. “Physicochemical Properties and Film‐Forming Ability of Fish Skin Collagen Extracted From Different Freshwater Species.” Process Biochemistry 50, no. 1: 148–155.

[fsn370059-bib-0056] Thuy, N. T. , and L. D. Minh . 2012. “Research Article Size Effect on the Structural and Magnetic Properties of Nanosized Perovskite LaFeO3 Prepared by Different Methods.”

[fsn370059-bib-0057] Truong, T. M. T. , V. M. Nguyen , T. T. Tran , and T. M. T. Le . 2021. “Characterization of Acid‐Soluble Collagen From Food Processing by‐Products of Snakehead Fish (*Channa striata*).” Processes 2021, no. 9: 1188. Extraction and Fractionation Processes of Functional Components in Food Engineering, 65.

[fsn370059-bib-0058] Turkish Statistical Institute . 2020. “Aquaculture Production.” https://www.tuik.gov.tr/PreTablo.do?alt_id=1043.

[fsn370059-bib-0059] Turkish Statistical Institute—TUIK . 2022. Su Ürünleri Istatistikleri. TUIK.

[fsn370059-bib-0060] Tziveleka, L. A. , E. Ioannou , D. Tsiourvas , P. Berillis , E. Foufa , and V. Roussis . 2017. “Collagen From the Marine Sponges *Axinella cannabina* and *Suberites carnosus*: Isolation and Morphological, Biochemical, and Biophysical Characterization.” Marine Drugs 15, no. 6: 152. 28555046 10.3390/md15060152 PMC5484102

[fsn370059-bib-0061] Tziveleka, L. A. , S. Kikionis , L. Karkatzoulis , K. Bethanis , V. Roussis , and E. Ioannou . 2022. “Valorization of Fish Waste: Isolation and Characterization of Acid‐and Pepsin‐Soluble Collagen From the Scales of Mediterranean Fish and Fabrication of Collagen‐Based Nanofibrous Scaffolds.” Marine Drugs 20, no. 11: 664. 36354987 10.3390/md20110664 PMC9697972

[fsn370059-bib-0062] Wang, H. , Y. Liang , H. Wang , H. Zhang , M. Wang , and L. Liu . 2014. “Physical‐Chemical Properties of Collagens From Skin, Scale, and Bone of Grass Carp (*Ctenopharyngo donidellus*).” Journal of Aquatic Food Product Technology 23, no. 3: 264–277.

[fsn370059-bib-0063] Wijaya, A. 2021. “Fish Bone Collagen.” Asian Journal of Fisheries and Aquatic Research 11, no. 6: 33–39.

[fsn370059-bib-0064] Woo, J. W. , S. J. Yu , S. M. Cho , Y. B. Lee , and S. B. Kim . 2008. “Extraction Optimization and Properties of Collagen From Yellowfin Tuna (*Thunnus albacares*) Dorsal Skin.” Food Hydrocolloids 22, no. 5: 879–887.

[fsn370059-bib-0065] Xu, Q. , J. Wang , and X. Li . 2018. “Collagen Extraction From Fish Skin Using Pepsin.” Food Chemistry 246: 356–362.

[fsn370059-bib-0066] Xu, S. , M. Gu , K. Wu , and G. Li . 2019. “Unraveling the Role of Hydroxyproline in Maintaining the Thermal Stability of the Collagen Triple Helix Structure Using Simulation.” Journal of Physical Chemistry B 123, no. 36: 7754–7763. 31418574 10.1021/acs.jpcb.9b05006

[fsn370059-bib-0067] Zhang, J. , R. Duan , Y. Tian , and K. Konno . 2009. “Characterisation of Acid‐Soluble Collagen From Skin of Silver Carp (*Hypophthalmichthys molitrix*).” Food Chemistry 116, no. 1: 318–322.

[fsn370059-bib-0068] Zhang, J. , R. Duan , C. Ye , and K. Konno . 2010. “Isolation and Characterization of Collagens From Scale of Silver Carp (*Hypophthalmichthys molitrix*).” Journal of Food Biochemistry 34, no. 6: 1343–1354.

